# Tunable activation of therapeutic platelet-rich plasma by pulse electric field: Differential effects on clot formation, growth factor release, and platelet morphology

**DOI:** 10.1371/journal.pone.0203557

**Published:** 2018-09-26

**Authors:** Andrew L. Frelinger, Anja J. Gerrits, V. Bogdan Neculaes, Thomas Gremmel, Andrew S. Torres, Anthony Caiafa, Sabrina L. Carmichael, Alan D. Michelson

**Affiliations:** 1 Center for Platelet Research Studies, Dana-Farber/Boston Children’s Cancer and Blood Disorders Center, Harvard Medical School, Boston, Massachusetts, United States of America; 2 GE Global Research Center, Niskayuna, New York, United States of America; 3 Department of Internal Medicine II, Medical University of Vienna, Vienna, Austria; Monash University, AUSTRALIA

## Abstract

**Background:**

Activation of platelet-rich plasma (PRP) by pulse electric field (PEF) releases growth factors which promote wound healing (*e*.*g*., PDGF, VEGF for granulation, EGF for epithelialization).

**Aims:**

To determine after PEF activation of therapeutic PRP: 1) platelet gel strength; 2) profile of released growth factors; 3) alpha- and T-granule release; 4) platelet morphology.

**Methods:**

Concentrated normal donor PRP was activated by 5 μsec (long) monopolar pulse, ~4000 V/cm (PEF A) or 150 nsec (short) bipolar pulse, ~3000 V/cm (PEF B) in the presence of 2.5 mM (low) or 20 mM (high) added CaCl_2_. Clot formation was evaluated by thromboelastography (TEG). Surface exposure of alpha granule (P-selectin) and T-granule (TLR9 and protein disulfide isomerase [PDI]) markers were assessed by flow cytometry. Factors in supernatants of activated PRP were measured by ELISA. Platelet morphology was evaluated by transmission electron microscopy (TEM).

**Results:**

Time to initial clot formation was shorter with thrombin (<1 min) than with PEF A and B (4.4–8.7 min) but clot strength (elastic modulus, derived from TEG maximum amplitude) was greater with PEF B than with either thrombin or PEF A (p<0.05). Supernatants of PRP activated with PEF A had higher EGF levels than supernatants from all other conditions. In contrast, levels of PF4, PDGF, and VEGF in supernatants were not significantly different after PEF A, PEF B, and thrombin activation. T-granule markers (TLR9 and PDI) were higher after thrombin than after PEF A or B with low or high CaCl_2_. By TEM, platelets in PEF-treated samples retained a subset of granules suggesting regulated granule release.

**Conclusion:**

Pulse length and polarity can be modulated to produce therapeutic platelet gels as strong or stronger than those produced by thrombin, and this is tunable to produce growth factor profiles enhanced in specific factors important for different stages of wound healing.

## Introduction

Platelet secretory granules contain large amounts of proteins and growth factors which are known to have various beneficial effects on wound healing such as angiogenesis and tissue regeneration [1,2]. Because of this, platelet-rich plasma (PRP) is frequently used as an autologous source of growth factors and cytokines to enhance wound healing [2,3], induce hemostasis [4], and provide antibacterial protection for the wound as it heals [5]. However, because wounds differ based on the type of injury (*e*.*g*., diabetic ulcer [6], burn [7]) and the stage of healing (*e*.*g*., granulation, angiogenesis, contraction, epithelialization [8,9]), and growth factors differ in their ability to influence these processes, it this unclear whether the balance of growth factors released by standard methods of platelet activation is optimal for all types and stages of wounds. Different growth factors appear to be selectively sorted into sub-populations of granules [10], raising the possibility that differential or selective release of subsets of granules could produce growth factor mixtures with improved activity in specific wound healing settings.

In addition to platelet alpha granules, dense granules, lysosomes and endosomes, a new type of platelet granule has been more recently described by Thon *et al*. [11]. These so-called T-granules are unique electron-dense, membrane-delimited intracellular compartments in platelets and co-localize with the toll-like receptor (TLR) 9 and protein disulfide isomerase (PDI), making these proteins markers for T-granules.

In our previous work [12–16] we demonstrated platelet activation via pulse electric field (PEF) stimulation using different electric pulses but at one CaCl_2_ concentration (10 mM final concentration). In this paper, we study platelet activation by 5 μsec (long) monopolar pulse, ~4000 V/cm (PEF A) or 150 nsec (short) bipolar pulse, ~3000 V/cm (PEF B) at two CaCl_2_ concentrations (2.5 mM and 20 mM). PEF B is the platelet activation using capacitive coupling (see [15]) via bipolar pulses, while PEF A uses extended duration monopolar pulses [14] in conductive coupling. Here we show that modulation of PEF conditions causes differential platelet release of growth factors, alters clot formation kinetics and strength, and is accompanied by alterations in platelet T-granule markers and platelet morphology.

## Methods

### Donors, blood collection and preparation of PRP

This study was conducted in compliance with the ethical principles of the Declaration of Helsinki. This study was reviewed and approved by the Boston Children’s Hospital Committee on Clinical Investigation (protocol # AX-09-0503-4) and all subjects provided written informed consent. Healthy volunteers were allowed to enroll in the study if they were aged ≥18 years, free of aspirin or other antiplatelet medication for ≥10 days, and free of all other non-steroidal anti-inflammatory drugs for ≥ 3 days. Following a 2 mL discard, 120 mL of blood was collected from each of 5 volunteers into 1/10^th^ volume of acid-citrate-dextrose solution A (ACD-A). PRP was prepared as previously described [15] according to the manufacturer’s recommendation using the Harvest^®^ SmartPreP2 System (Harvest Technologies, Plymouth, MA, USA) with two 60 mL cartridges. The resultant PRP was pooled prior to further treatment. Complete blood cell counts were performed on the ACD-anticoagulated whole blood and the concentrated PRP in a Sysmex XN Hematology Analyzer.

### Study design

PRP activation by PEF (conditions described below) and bovine thrombin (1 U/mL final concentration, Biopharm Laboratories LLC, Bluffdale, UT, USA) were evaluated in parallel in the presence of low (2.5 mM) and high (20 mM) concentrations of CaCl_2_. Controls included PRP treated with vehicle (saline) with or without 20 mM CaCl_2_. Endpoints measured included 1) clot formation kinetics and strength by thromboelastography (TEG) and prothrombin fragment F1.2 generation, 2) platelet surface expression of alpha granule membrane (identified by P-selectin) and T-granule membrane (identified by TLR9, and PDI) markers measured by flow cytometry, 3) growth factors released from platelets into the supernatant (endothelial growth factor [EGF], platelet-derived growth factor [PDGF], vascular endothelial growth factor [VEGF], platelet factor 4 [PF4], PDI), and 4) platelet morphology by transmission electron microscopy (TEM). All endpoints except TEG were measured in samples taken 15 min after activation.

Prior to activation with PEF or thrombin, PRP samples were recalcified by addition of 1/100^th^ volume of CaCl_2_ (2.5 mM or 20 mM final concentrations, Bachem, Torrance, CA, USA). A total of 2 mL of concentrated PRP was treated under each condition. For analysis by TEG, 360 μL of activated PRP was quickly transferred to the TEG cup and recordings initiated immediately. A small portion of the activated PRP (18 μL) was used to assess platelet surface expression of alpha granule membrane (P-selectin) and T-granule membrane (TLR9 and PDI) markers by flow cytometry. This aliquot was mixed with Gly-Pro-Arg-Pro (GPRP, 2 μL, 40 mM final concentration, Bachem) to prevent clotting, stained for 15 min with a cocktail of fluorescent antibodies as described in detail below, then fixed by addition of 1% formaldehyde. Another portion (40 μL) of the activated PRP was fixed 15 minutes after activation and further processed for analysis by TEM. The remainder of the sample was allowed to stand 15 min at RT following activation, then clots were removed using the wooden handle of a cotton swab and the resulting serum was frozen at -80° C for later evaluation of released growth factors.

### Pulse electric field stimulation of PRP

**Fig 1:**
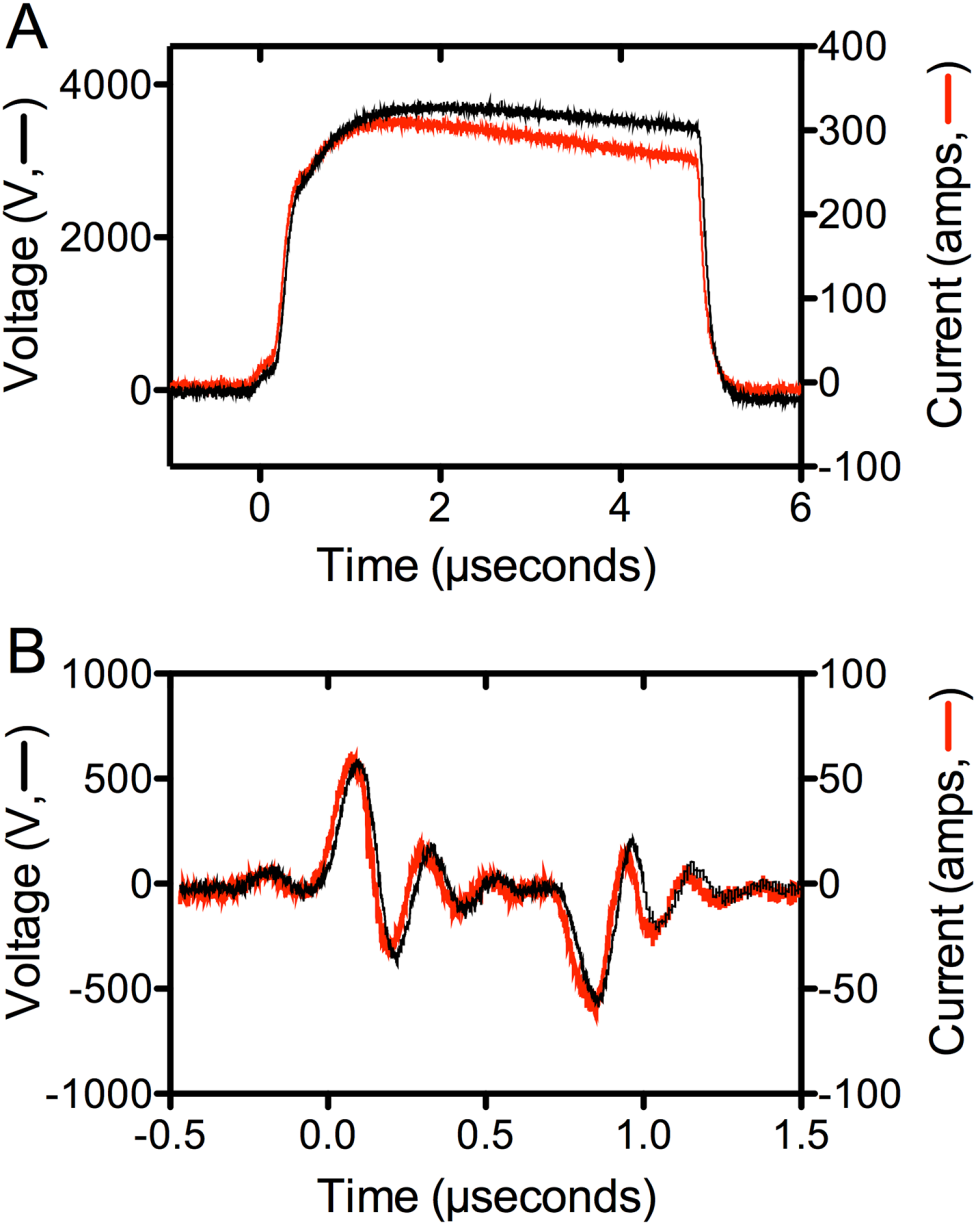
Representative electrical tracings for PEF A (A) and PEF B (B).

Electrical stimulation of PRP was performed using a specially designed instrument prototype (GE Global Research, Niskayuna, NY, USA), which has previously been described [12,15]. Concentrated PRP (500 μL) was placed in a 2 mm electroporation cuvette (Molecular BioProducts, San Diego, CA, USA), pre-loaded with 1/100^th^ volume CaCl_2_ (2.5 or 20 mM final concentration), and exposed to one of two conditions: PEF A (1 pulse,5 μsec pulse width, ~4000 V/cm) or PEF B (bipolar pulses, ~650 ns interval between pulses of opposite polarity, 150 ns pulse width, 600 V pulse amplitude / 3000 V/cm, 80 pairs of bipolar pulses, each pair delivered at one second) in the presence of 2.5 mM or 20 mM added CaCl_2_. As previously described [15] a Tektronix DPO4104 oscilloscope and a Tektronix P6015A high voltage probe were used to measure the voltage pulses applied to cuvettes with PRP for activation. Fig 1 shows representative electrical traces for blood stimulated with PEF A and PEF B.

### Thromboelastography and activation of plasma prothrombin

Immediately following exposure of PRP to activating conditions, 360 μL of treated PRP was placed in a TEG cup and analysis by a TEG 5000 Hemostasis Analyzer System (Haemonetics Corporation, Braintree, MA, USA) was started. Clotting kinetics and characteristics were followed for 30 minutes. Conversion of plasma prothrombin to thrombin was evaluated by measurement of prothrombin fragment F1.2 by ELISA (Enzygnost, Siemens, Marburg, Germany), according to the distributor’s manual, in supernatants collected 15 min after PRP activation.

### Platelet alpha granule and T-granule release

Flow cytometry was used to assess differential release of platelet granules and granule contents as measured by changes in platelet surface P-selectin (for alpha granules) and platelet surface PDI and TLR9 (for T-granules), as previously described [11,17,18]. Briefly, samples for flow cytometry were fixed 15 min after activation by addition of an equal volume of 2% formaldehyde in 10 mM HEPES, 0.15 M NaCl, pH 7.4. Samples were diluted 12-fold in HEPES-saline buffer (10 mM HEPES, 0.15 M NaCl, pH 7.4; chemicals from Sigma, St. Louis, MO, USA) then added to a mixture of fluorescein isothiocyanate (FITC)-conjugated anti-TLR9 (clone 5G5, Abcam, Cambridge, MA, USA), phycoerythrin (PE)-conjugated P-selectin (clone AK4, BD Pharmingen, San Diego, CA, USA) and CD41-PerCP-Cy5.5 (clone HIP8, BD Pharmingen, San Diego, CA, USA) or to a mixture of FITC-conjugated anti-P-selectin (clone AK4, BD Pharmingen, San Diego, CA, USA), PE-conjugated PDI (clone 1D3, Abcam, Cambridge, MA, USA) and CD41-PerCP-Cy5.5. Non-specific staining was determined in parallel using a sample reacted with a mixture of isotype-matched FITC, isotype-matched PE and CD41-PerCP-Cy5.5. After 30 minutes of staining at room temperature, 400 μL of 1% formaldehyde in HEPES-saline buffer was added. Flow cytometric analysis was performed in a calibrated Becton Dickinson FACSCalibur.

T-granule release was further evaluated by measurement of soluble PDI by ELISA (Cloud-Clone Corp., Houston, TX, USA), according to the distributor’s manual, in supernatants collected 15 min after PRP activation.

### Growth factor release

Levels of EGF, PDGF, VEGF and PF4 in the supernatants of the treated PRP were measured using commercially available ELISA kits (EGF and PDGF, R&D Systems, Minneapolis, MN, USA; VEGF, Eagle Biosciences, Nashua, NH, USA; PF4, Abcam, Cambridge, UK).

### Transmission electron microscopy

Treated PRP (40 μL) was fixed at 15 min after activation with 40 μL of 2.5% glutaraldehyde, 1.25% paraformaldehyde and 0.03% picric acid in 0.1 M sodium cacodylate buffer, pH 7.4 and stored at 4°C until preparation for TEM. After fixation, samples were washed in 0.1 M cacodylate buffer and post-fixed with 1% osmium tetroxide (OsO_4_)/1.5% potassium ferrocyanide (KFeCN_6_) for 1 hour, washed in water three times, and incubated in 1% aqueous uranyl acetate for 1 hour followed by 2 washes in water and subsequent dehydration (10 min each) in 50%, 70%, 90% and 100% alcohol. Following an additional 10 min dehydration in 100% alcohol, samples were then put in propyleneoxide for 1 hour and infiltrated overnight in a 1:1 mixture of propyleneoxide and TAAB Epon (Marivac Canada Inc., St. Laurent, Canada). The following day the samples were embedded in TAAB Epon and polymerized at 60 degrees C for 48 hrs. Ultrathin sections (about 60 nm) were cut on a Reichert Ultracut-S microtome, picked up onto copper grids, stained with lead citrate and examined in a Tecnai G^2^ Spirit BioTWIN transmission electron microscope. Images were recorded with an AMT 2k CCD camera. Processing and TEM imaging was performed by the Harvard Medical School electron microscopy core facility (Boston, MA, USA).

### Statistical analysis

Data were analyzed using GraphPad Prism version 5.0a (GraphPad Software, La Jolla, CA, USA). Normally distributed data (as judged by the D’Agostino and Pearson omnibus normality test) are summarized as mean ± standard deviation or mean ± standard error of the mean, as indicated. Non-parametric data are reported as median and interquartile range or median and range. One way ANOVA was used for comparison of three or more groups, with Tukey’s multiple comparison post-test for individual comparisons.

## Results

### Preparation of PRP

The platelet count of PRP prepared from fresh blood of 5 healthy volunteers using a clinically relevant procedure [19] was 1193 ± 336 x 10^9^/L (mean ± SD), a > 4-fold increase compared to the platelet count found in whole blood (275 ± 74 x 10^9^/L, Table 1). The WBC count in PRP was 3-fold more than whole blood, while hematocrit and RBC count were about half those in whole blood (Table 1).

**Table 1:** Cell composition and fold concentration of PRP prepared using the Harvest system.

Parameter	Whole Blood	PRP	Fold-Concentration of PRP Compared to Whole Blood
WBC (×10^9^/L)	4.63 ± 0.91	13.79 ± 2.44	3.00 ± 0.33
RBC (×10^12^/L)	4.14 ± 0.48	2.02 ± 0.39	0.49 ± 0.07
HCT (%)	38.2 ± 3.5	20.4 ± 2.9	0.52 ± 0.09
PLT (×10^9^/L)	275 ± 74	1193 ± 336	4.33 ± 0.19

Abbreviations: HCT, hematocrit; PLT, platelet; PRP, platelet-rich plasma; RBC, red blood cell; WBC, white blood cell. Data are mean ± SD, n = 5.

### Clot formation characteristics

**Fig 2:**
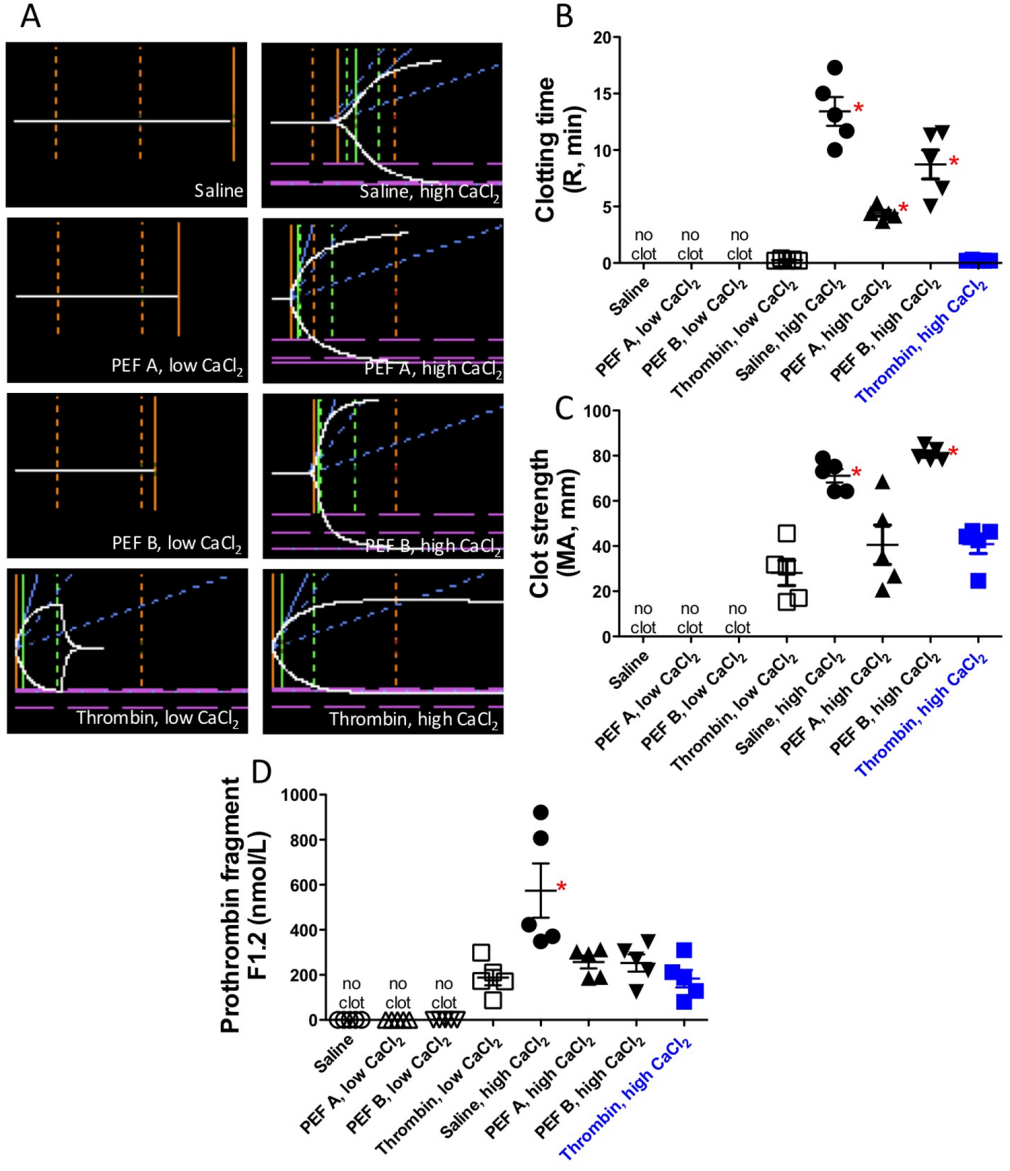
Characteristics of clot formation following exposure of PRP to PEF or thrombin at low (2.5 mM) and high (20 mM) concentrations of CaCl_2_. A) Representative TEG plots, B) clotting time (R in min), C) clot strength (maximum amplitude [MA] in mm), D) prothrombin fragment F.1.2 (nmol/L). Individual results are plotted as mean ± SEM (n = 5). Asterisks indicate p<0.05 *vs*. thrombin, high CaCl_2_ (filled blue squares) by Dunnett’s multiple comparison test (following ANOVA).

Clotting kinetics and clot strength were evaluated by transferring a portion of the treated PRP to TEG cups and initiating recordings immediately after activation with the following conditions: 1) PEF A/low CaCl_2_, 2) PEF B/low CaCl_2_, 3) PEF A/high CaCl_2_, 4) PEF B/high CaCl_2_, 5) 1 U/mL bovine thrombin/low CaCl_2_, 6) 1 U/mL bovine thrombin/high CaCl_2_, 7) 10 mM HEPES/0.15 M NaCl, 8) 10 mM HEPES/0.15 M NaCl/high CaCl_2_. Time to initial clot formation, R, was shortest for thrombin treated samples, occurring in less than a minute (0.2 ± 0.0 for high CaCl_2_ [in min], whereas PEF A high CaCl_2_ required 4.4 ± 0.6 min, PEF B high CaCl_2_ required 8.7 ± 2.9 min, and high CaCl_2_ alone initiated clots in 13.4 ± 2.8 minutes (Fig 2A, Table 2). Despite taking more time for initial clot formation to occur, clot strength (MA) was highest in the PEF B/high CaCl_2_ sample and the CaCl_2_ alone control. Clot strength (maximum amplitude: MA) was similar for thrombin/high CaCl_2_ and PEF A/high CaCl_2_, although clot strength appeared to be more variable with PEF A/high CaCl_2_. Clots were not detected by TEG up to 30 min after activation with PEF A/low CaCl_2_ or PEF B/low CaCl_2_ or treatment with buffer, no CaCl_2_ (Fig 2B, Table 2).

**Table 2:** Comparison of PEF conditions and CaCl_2_ levels *vs*. bovine thrombin on clotting kinetics, clot strength and thrombin formation.

	PEF A, low CaCl_2_	PEF B, low CaCl_2_	PEF A, high CaCl_2_	PEF B, high CaCl_2_	Thrombin, low CaCl_2_	Thrombin, high CaCl_2_	Saline	Saline, high CaCl_2_
TEG R (min)	no clot	no clot	4.4 ± 0.6*	8.7 ± 2.9*	0.3 ± 0.1	0.2 ± 0.0	no clot	13.4 ± 2.8*
TEG MA (mm)	no clot	no clot	40.6 ± 19.5	80.7 ± 3*	28.1 ± 12.4	40.9 ± 9.3	no clot	71.1 ± 6.6*
F1.2 (nmol/L)	0.3 ± 0.2	0.3 ± 0.2	257 ± 63.1†	252.9 ± 85.7†	187.9 ± 76.4†	183.5 ± 87†	0.1 ± 0.1	574.1 ± 269.2*†

* p<0.05 *vs*. thrombin, high CaCl_2_;

^†^ p<0.05 *vs*. saline.

Abbreviations: MA, maximum amplitude; R, time of latency from start of test to initial fibrin formation; TEG, thromboelastogram. Mean ± SD, n = 5

To determine the degree to which the CaCl_2_, PEF, thrombin, and control conditions contributed to conversion of plasma prothrombin to active thrombin, F1.2 was measured in the supernatant (Fig 2C, Table 2). The highest levels of F1.2 were observed for PRP incubated with high CaCl_2_ alone, while F1.2 was undetectable for the no CaCl_2_ control and for both PEF A/low CaCl_2_ and B/low CaCl_2_. These results are consistent with the results of the TEG studies, which showed no detectable clotting in the absence of CaCl_2_ or after 30 minutes treatment with PEF A/low CaCl_2_ and PEF B/low CaCl_2_ (Fig 2A and 2B).

### Differential exposure of platelet alpha granule and T granule markers

**Fig 3:**
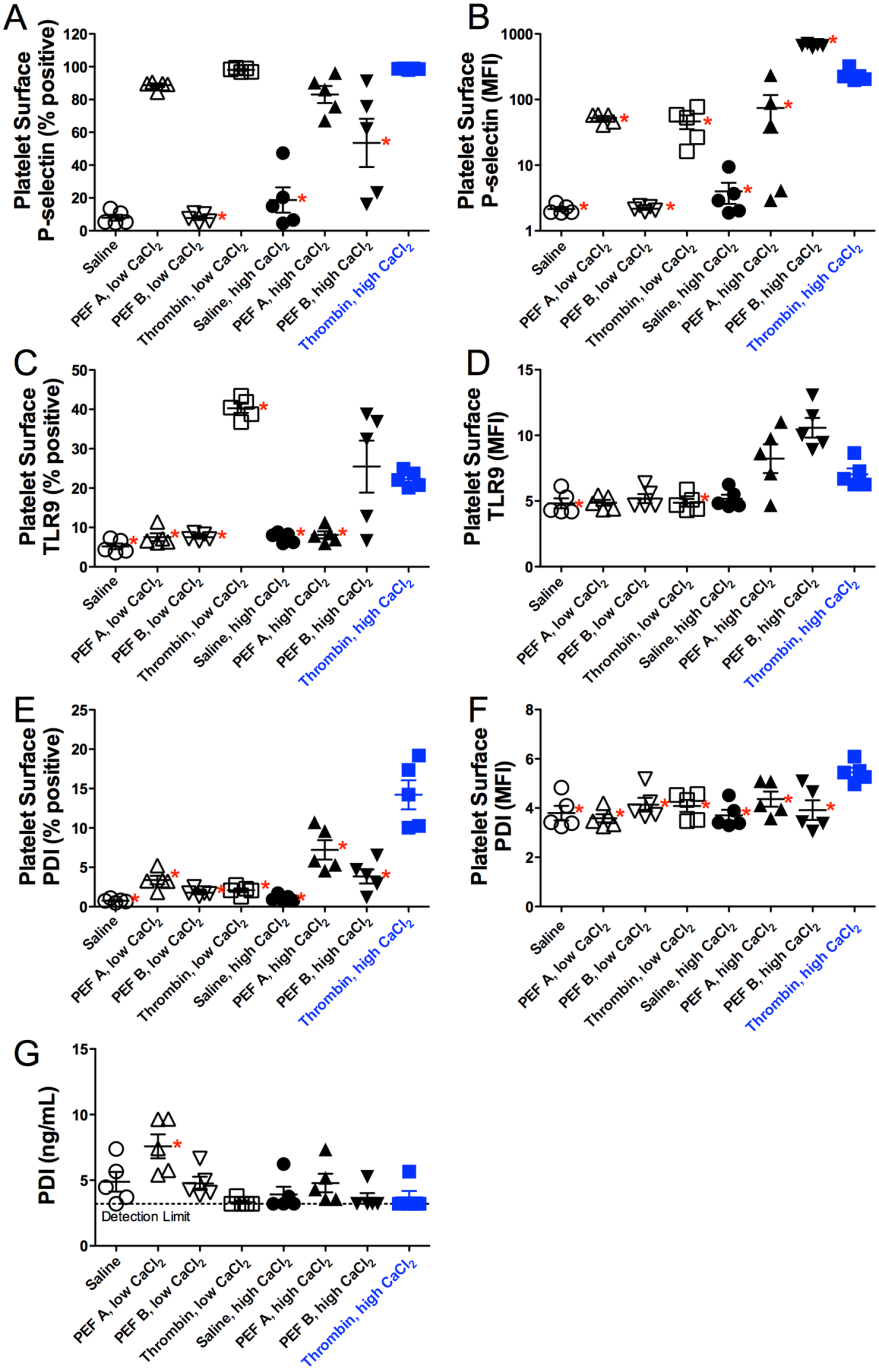
Platelet surface markers of degranulation for alpha granules (P-selectin) and T granules (TLR9 and PDI), and soluble PDI, following exposure of PRP to PEF, thrombin, or control conditions. Individual results are plotted as mean ± SEM. Asterisks indicate p<0.05 *vs*. thrombin, high CaCl_2_ (filled blue squares) by Dunnett’s multiple comparison test (following ANOVA). MFI, mean fluorescence intensity.

Following activation of PRP, a portion of each sample was mixed with the peptide Gly-Pro-Arg-Pro (GPRP) which prevents fibrin polymerization into clots, thereby allowing flow cytometric analysis of platelet surface markers by flow cytometry [20]. Release of platelet alpha granules was monitored by exposure of platelet surface P-selectin (CD62P), while release of platelet T granules was monitored by exposure of platelet surface TLR9 and PDI (Fig 3, Table 3). Greater than 80 percent of platelets were positive for P-selectin following treatment with thrombin/low or high CaCl_2_ and PEF A/low or high CaCl_2_, while a lower percentage (53.6 ± 33, mean ± SD) of platelets were P-selectin-positive with PEF B/high CaCl_2_. High CaCl_2_ alone caused a modest increase in the percent P-selectin-positive platelets compared to PEF B/low CaCl_2_ and the no CaCl_2_ control (18.8 ± 17.2 *vs*. 7.8 ± 2.7 and 8.0 ± 3.9 respectively) (Fig 3A, Table 3). In contrast with our previous studies [13], the mean fluorescence intensity of P-selectin per particle with PEF treatment was lower than that seen with thrombin and low or high CaCl_2_ (Fig 3B).

**Table 3:** Platelet surface markers P-selectin, TRL9, PDI and platelet-derived PDI following exposure of PRP to PEF, thrombin, or control conditions.

	PEF A, low CaCl_2_	PEF B, low CaCl_2_	PEF A, high CaCl_2_	PEF B, high CaCl_2_	Thrombin, low CaCl_2_	Thrombin, high CaCl_2_	Saline	Saline, high CaCl_2_
Platelet surface P-selectin (% pos)	88.7 ± 2.4†	7.8 ± 2.7*	83.0 ± 11.6†	53.6 ± 32.8*†	98.0 ± 1.2†	98.6 ± 0.4†	8.0 ± 3.9*	18.8 ± 17.2*†
Platelet surface P-selectin (MFI)	52.4 ± 8.5*	2.2 ± 0.2*	46.4 ± 24.7*	74.2 ± 96.3*	663.8 ± 36*†	235.6 ± 50.1†	2.1 ± 0.3*	4.0 ± 3.1*
Platelet surface TLR9 (% pos)	7.5 ± 2.2*	7.5 ± 0.9*	8.1 ± 2*	25.5 ± 14.7†	40.3 ± 2.6*†	22.3 ± 2.0†	5.2 ± 1.7*	7.5 ± 1.3*
Platelet surface TLR9 (MFI)	4.9 ± 0.5	5.2 ± 0.8	4.9 ± 0.6	8.2 ± 2.5†	10.6 ± 1.7*†	7.0 ± 1.0†	4.8 ± 0.9*	5.2 ± 0.7
Platelet surface PDI (% pos)	3.4 ± 1.2*	1.8 ± 0.4*	7.2 ± 2.8*†	3.8 ± 2*	2.1 ± 0.5*	14.2 ± 4.1†	0.8 ± 0.2*	1.1 ± 0.4*
Platelet surface PDI (MFI)	3.6 ± 0.4*	4.1 ± 0.6*	4.1 ± 0.5*	4.4 ± 0.7*	3.9 ± 0.9*	5.5 ± 0.4†	3.8 ± 0.7*	3.7 ± 0.5*
Platelet-derived PDI (ng/mL)	7.6 ± 2*†	4.8 ± 1.1	4.8 ± 1.6	3.6 ± 0.9	3.3 ± 0.3	3.7 ± 1.1	4.9 ± 1.7	3.9 ± 1.3

* p<0.05 *vs*. thrombin, high CaCl_2_;

^†^ p<0.05 *vs*. saline.

Abbreviations: MFI, mean fluorescence intensity; PDI, protein disulfide isomerase; TLR9, toll-like receptor 9; mean ± SD.

Platelet surface TLR9 was highest with thrombin/low CaCl_2_. Lower TLR9 and highly variable levels of TLR9 were observed for PEF B/high CaCl_2_, and negligible TLR9 expression was observed for PEF A/low and high CaCl_2_ and B/low CaCl_2_ and for the no CaCl_2_ and high CaCl_2_ only controls. Overall, while the levels of TLR9 were lower than P-selectin, the pattern of TLR9 expression was not distinctly different from that seen for P-selectin (Fig 3C and 3D). In contrast, the pattern of PDI expression was unique in that the highest levels were seen with thrombin/high CaCl_2_ instead of thrombin/low CaCl_2_ (Fig 3E and 3F). T granule release was also estimated by release of PDI into the supernatants of treated PRP samples as measured by ELISA (Fig 3G, Table 3). PDI concentrations were greatest in the supernatants of PRP activated with PEF A/low CaCl_2_ while levels were near or below the detection limit of the assay for samples treated with thrombin (Fig 3G). This contrasts with platelet surface PDI measured by flow cytometry, which was highest with thrombin high CaCl_2_ (Fig 3E and 3F). Thus, there appear to be significant differences between activating conditions with respect to free *vs*. surface bound PDI.

### Differential release of platelet granules and growth factors

**Fig 4:**
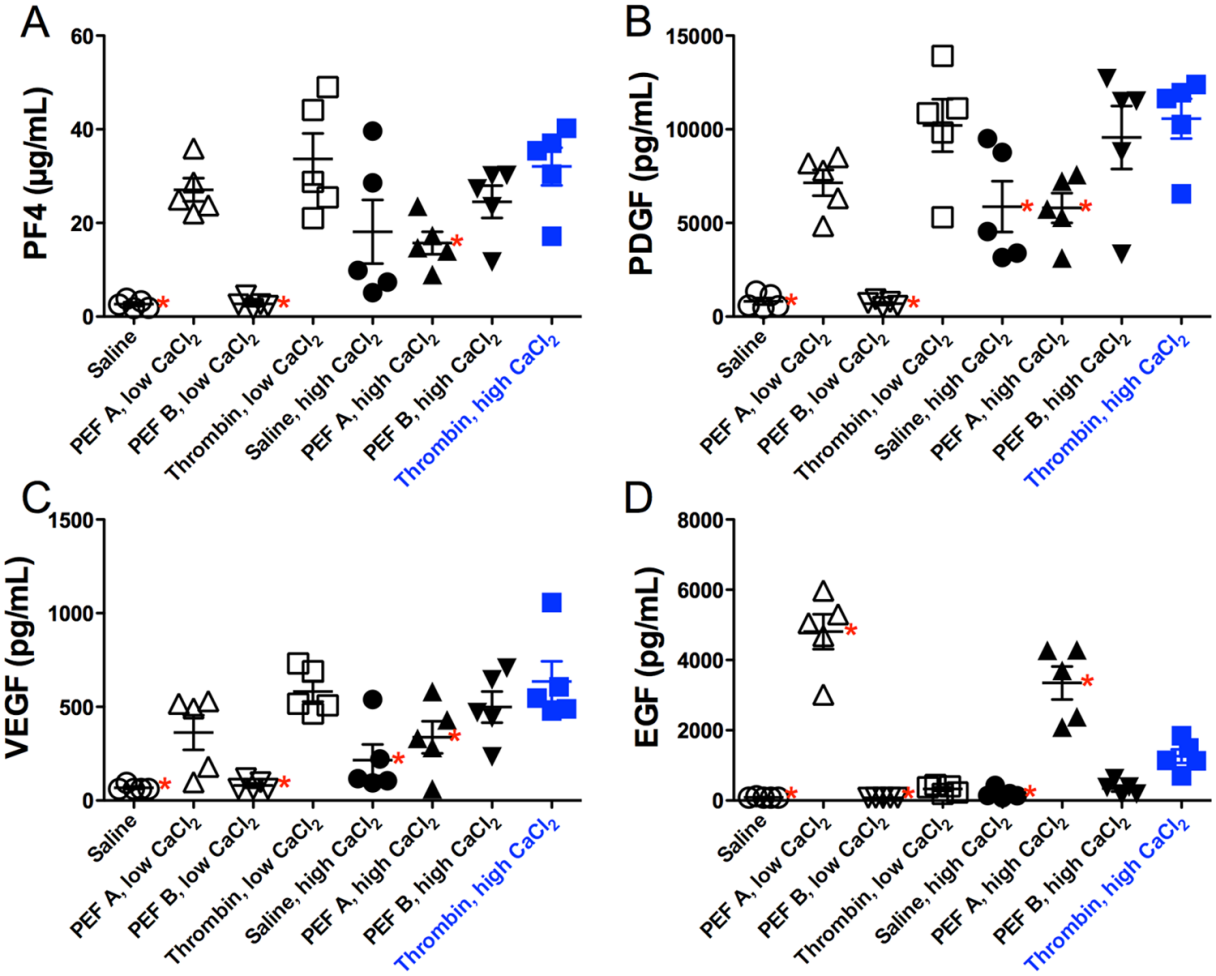
Growth factors present in the supernatant following PEF, thrombin, or control treatment of PRP at low and high CaCl_2_. Results below the detection limit of the assay are plotted at the lower limit of detection. Individual results are plotted as mean ± SEM. Asterisks indicate p<0.05 *vs*. thrombin, high CaCl_2_ (filled blue squares) by Dunnett’s multiple comparison test (following ANOVA).

**Fig 5:**
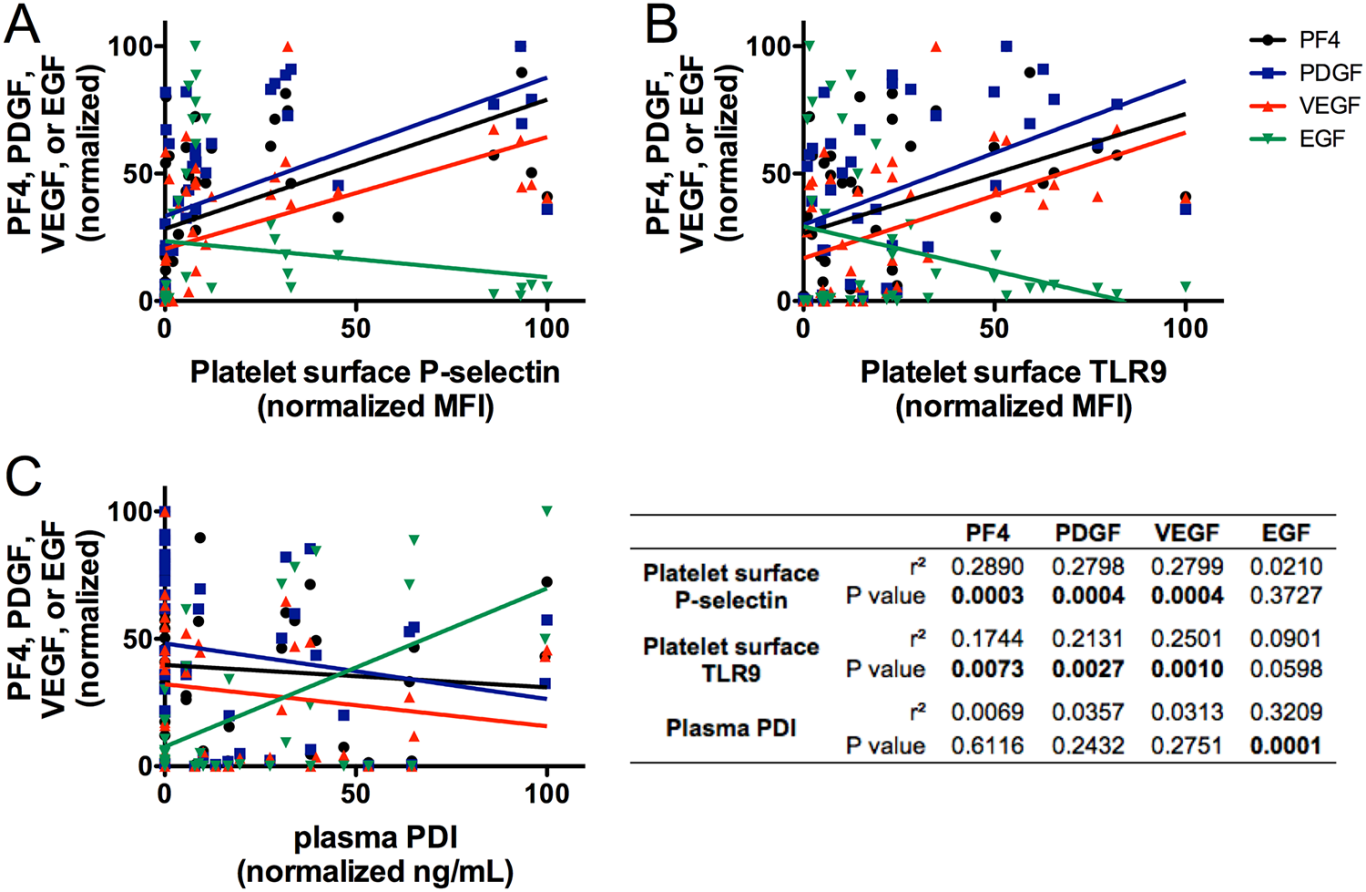
Correlation of granule markers with growth factor release.

The presence of growth factors in supernatants after treatment of PRP with PEF, thrombin, or control conditions was evaluated by ELISA (Fig 4, Table 4). The amount of growth factor released by each treatment condition varied widely but the overall pattern of PF4, PDGF, and VEGF release by treatment was similar. Specifically, the relative ability of the treatment conditions to increase amounts of PF4, PDGF, and VEGF in supernatants of PRP were, from most to least, thrombin/high CaCl_2_ = thrombin/low CaCl_2_ > PEF B/high CaCl_2_ = PEF A/low CaCl_2_ > PEF A/high CaCl_2_, > high CaCl_2_ alone, > PEF B/low CaCl_2_ = no CaCl_2_ (Fig 4). In contrast, conditions that yielded the most to least release of EGF were PEF A/low CaCl_2_ >> PEF A/high CaCl_2_, >> thrombin/high CaCl_2_ > thrombin/low CaCl_2_ = PEF B/high CaCl_2_, = high CaCl_2_ alone > PEF B/low CaCl_2_, = no CaCl_2_ (Fig 4). Consequently, levels of PF4, PDGF, and VEGF were highly correlated with one another while EGF levels were not correlated with PF4, PDGF, and VEGF (Table 5). Interestingly, soluble PDI levels were best correlated with released EGF levels (r = 0.566, p = 0.0001) (Fig 5).

**Table 4:** Growth factors in supernatant of activated PRP. Results below the lower limit of detection are reported using the value of the lower limit (VEGF, 62 pg/mL; EGF 78 pg/mL).

	PEF A, low CaCl_2_	PEF B, low CaCl_2_	PEF A, high CaCl_2_	PEF B, high CaCl_2_	Thrombin, low CaCl_2_	Thrombin, high CaCl_2_	Saline	Saline, high CaCl_2_
PF4 (μg/mL)	27.1 ± 5.5†	2.7 ± 1.1*	15.7 ± 5.3*	24.5 ± 7.7†	33.7 ± 12.2†	32.1 ± 9†	2.7 ± 0.9*	18.1 ± 15.2†
PDGF (μg/mL)	7.14 ± 1.53†	0.70 ± 0.17*	5.80 ± 1.77*†	9.56 ± 3.77†	10.21 ± 3.13†	10.56 ± 2.38†	0.82 ± 0.4*	5.87 ± 3.04*†
VEGF (pg/mL)	363.4 ± 207†	80.2 ± 26*	337.7 ± 191.7*	499 ± 186.5†	582.3 ± 120.3†	635.6 ± 241†	68.5 ± 14.6*	215.6 ± 188.1*
EGF (ng/mL)	4.81 ± 1.11*†	0.08 ± 0.00*	3.35 ± 1.05*†	0.34 ± 0.19	0.33 ± 0.11	1.26 ± 0.43†	0.09 ± 0.02*	0.20 ± 0.14*

* p<0.05 *vs*. thrombin, high CaCl_2_;

^†^ p<0.05 *vs*. saline.

Abbreviations: EGF, epidermal growth factor; PDGF, platelet-derived growth factor; PF4, platelet factor 4; VEGF, vascular endothelial growth factor.

**Table 5:** Growth factor correlation matrix: Coefficients and p values.

	PF4		PDGF		VEGF		EGF	
	r	p	r	p	r	p	r	p
**PF4**			0.917	9.2 x 10^−17^	0.758	1.5 x 10^−8^	0.264	0.0998
**PDGF**	0.917	9.2 x 10^−17^			0.796	8.34 x 10^−10^	0.199	0.2179
**VEGF**	0.758	1.5 x 10^−8^	0.796	8.34 x 10^−10^			0.117	0.4728
**EGF**	0.264	0.0998	0.199	0.2179	0.117	0.4728		

Abbreviations: EGF, epidermal growth factor; PDGF, platelet-derived growth factor; PF4, platelet factor 4; VEGF, vascular endothelial growth factor.

### Platelet morphology by transmission electron microscopy (TEM)

**Fig 6:**
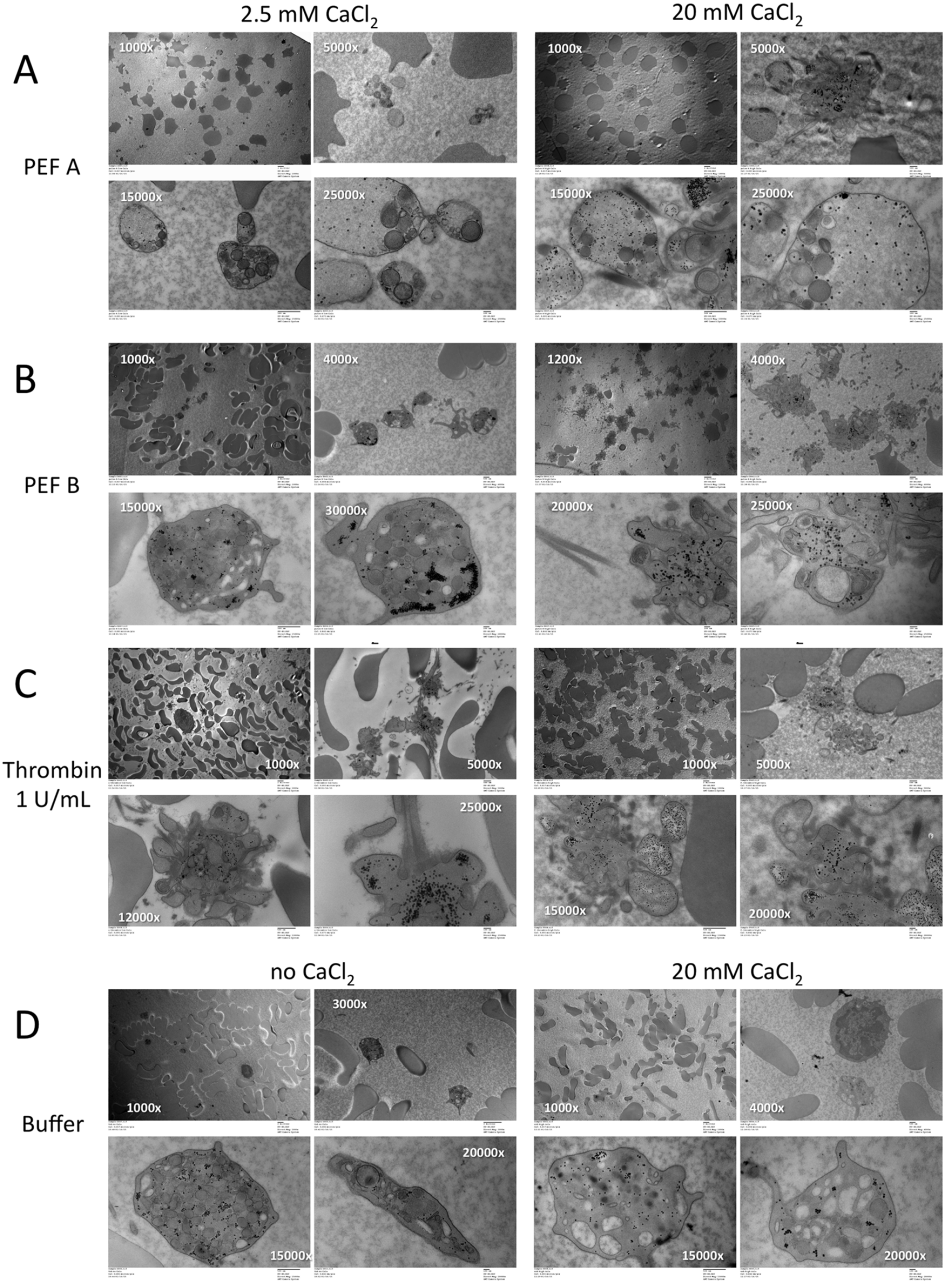
Platelet morphology by transmission electron microscopy following activation of PRP. Concentrated PRP supplemented with the indicated concentrations of CaCl_2_ were activated by A) PEF A, B) PEF B, C) bovine thrombin, or D) vehicle, then processed as described in Methods. Direct magnification is indicated. Representative images are shown for one representative donor.

TEM was used to evaluate platelet morphology and the presence of granules following activation of concentrated PRP with PEF, thrombin, or vehicle (Fig 6, S1–S8 Figs). Platelets in PRP treated with PEF A/low CaCl_2_ appeared swollen, round or ovoid and without filopodia or recognizable surface-connected open canalicular system (OCS) (Fig 6A, S1 Fig). Granules were detectable in these platelets and were asymmetrically distributed. Glycogen was reduced compared to unstimulated platelets and was dispersed fairly uniformly throughout the cytoplasm. Erythrocytes were irregularly shaped with spicules and did not show clumping or rouleaux formation. Similar results were observed for PEF A/high CaCl_2_ (Fig 6A, S2 Fig) but, in addition, platelet aggregates were observed as well as polymerized fibrinogen/fibrin. In contrast, platelets exposed to PEF B/low CaCl_2_ appeared grossly normal, discoid with a few extended filopodia, discernable OCS and normal granule and glycogen distribution (Fig 6B, S3 Fig). Erythrocytes were discoid shaped and exhibited rouleaux formation. However, following exposure of PRP to PEF B/high CaCl_2_, significant numbers of platelet aggregates, each containing 5–10 platelets were observed with some remaining granules and glycogen inclusions and with extended filopodia interacting with polymerized fibrinogen/fibrin strands (S4 Fig). Few erythrocytes were observed and those that were present appeared to be abnormally shaped or fragmented, suggesting significant hemolysis.

**Fig 7:**
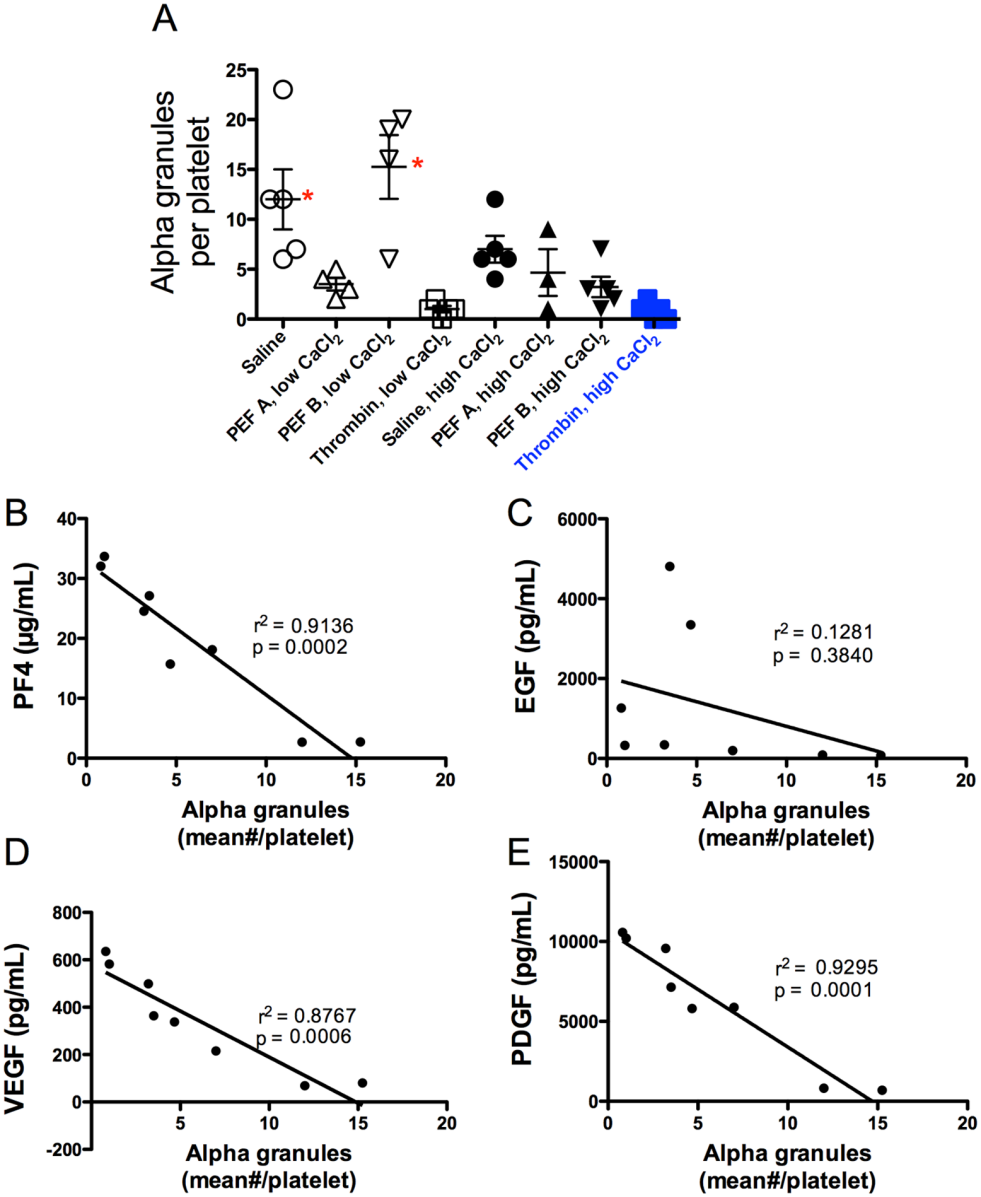
Platelet alpha granule content following activation of PRP and correlation with supernatant growth factor levels. A) Quantitation of alpha granules in TEM images of platelets following activation of PRP. Asterisks indicate p<0.05 *vs*. thrombin, high CaCl_2_ (filled blue squares) by Dunnett’s multiple comparison test (following ANOVA). B—E) Correlation of residual platelet alpha granule numbers with growth factor concentrations in supernatants following PEF, thrombin, or control treatment of PRP at low and high CaCl_2_.

Control PRP samples supplemented with low or high CaCl_2_ then treated with thrombin contained small clusters of aggregated platelets (2–5/aggregate) and individual activated platelets with extended filopodia associated with fibrinogen/fibrin strands (Fig 6C, S5 and S6 Figs). Few granules were discernable within the platelet. With thrombin/low CaCl_2_, the erythrocytes appeared as distorted discoid shapes whereas with thrombin/high CaCl_2_ their shape appeared normal and rouleaux formation was present. PRP treated with vehicle alone (no CaCl_2_ or agonist) showed the expected discoid platelet morphology and granule content and OCS distribution, although short filopodia were detected extending from many platelets (Fig 6D, S7 Fig). Likewise, erythrocytes showed normal morphology in vehicle-treated samples. However, platelets in PRP fixed for TEM 15 min after addition of high CaCl_2_, a time at which clotting was observed by TEG (Fig 1A), show a more irregular shape although granules, glycogen and OCS are still clearly present (Fig 6D, S8 Fig). Clustering of extracellular staining was suggestive of fibrinogen/fibrin strands, but thick fibrinogen/fibrin strands with their characteristic repeating pattern were not detected. Erythrocytes were a mixture of normal discoid shapes (individually and in rouleaux formation), distorted discs and irregular shapes with spicules. Fig 7A shows quantitation of alpha granules for each treatment. The mean residual number of alpha granules seen on TEM images was highly correlated with concentrations of PF4, VEGF and PDGF, but not EGF, in the supernatants of treated samples (Fig 7B–7E).

## Discussion

Activated PRP is used clinically to enhance wound healing [2,3,21], in part because of the growth factors it releases [22,23]. However, different growth factors are important at different stages of wound healing [8,9,24]. Thus, in the present study, we evaluated the potential of PEF activation of PRP to produce mixtures of growth factors enhanced for factors that target specific stages of wound healing. The main findings of this study are 1) PEF conditions can be modulated to produce platelet gels as strong as those produced by thrombin, with growth factor profiles enhanced in specific factors important for different stages of wound healing, 2) differences in the levels of EGF released relative to other growth factors suggests EGF distribution within platelets is different from other growth factors, 3) the reproducible pattern of growth factor release with PEF combined with the distinct morphology of PEF-treated platelets suggests PEF initiates a regulated release of platelet contents rather than non-specific mechanical fracturing. In addition, we have shown that the combination of PEF A and low CaCl_2_ triggers growth factor release but no clotting, a major difference from thrombin activation, that is accompanied by growth factor release and clotting. Therefore with PEF activation, growth factor release with or without clotting can be achieved in a controllable fashion.

### PRP

In order to ensure that the analyses performed would be relevant to current clinical practice, the PRP was prepared using a clinically available closed system instrument, the Harvest SmartPReP2. This instrument is currently used in many physician practices and provides sterile autologous PRP. Table 1 shows that while the RBC concentration is reduced by approximately half compared to whole blood, the PRP prepared by this method still contains high levels of RBCs which could potentially release ADP leading to platelet activation. However, following PRP preparation by this method, only 8.0 ± 3.9% (mean ± SD) of platelets were P-selectin positive and platelet surface P-selectin mean fluorescence intensity was only 2.1 ± 0.3 (Table 3) demonstrating that, despite the presence of high levels of RBCs, the PRP isolation procedure caused minimal platelet activation. Furthermore, the platelets in the PRP were not desensitized by ADP released from RBCs as evidenced by their ability to become fully activated in response to thrombin activation (98.6 ± 0.4% P-selectin positive, platelet surface P-selectin mean fluorescence intensity 235.6 ± 50.1 (mean ± SD), Table 3).

Table 1 also shows that the PRP prepared using the Harvest system contains leukocytes at three-fold higher concentration compared to their concentration in whole blood. Whether leukocytes are beneficial to the clinical efficacy of PRP is unclear.[25–28] However, they may contribute to the antimicrobial activity of PRP preparations.[29]

### Clot formation, strength and structure

Prior to application to a wound, PRP is currently converted to a gel either by addition of exogenous thrombin or activation of the thrombin present in the plasma. PEF activation of PRP results in platelet gel formation,[12] but whether thrombin plays a role in this process has not previously been investigated. Re-calcification of ACD-anticoagulated PRP with high CaCl_2_ results in an estimated free Ca^2+^ concentration of 5 mM (calculated based on the overall binding constant between citrate and Ca^2+^ [30]) and eventually results in thrombin generation as evidenced by F1.2 produced (Fig 2C) and the formation of a clot (Fig 2A and 2B) whose strength (elastic modulus, derived from TEG maximum amplitude) was greater than that of clots formed following direct addition of thrombin (Fig 2B). Adding PEF A or PEF B treatment immediately after re-calcification of PRP with high CaCl_2_ shortened the time required to initiate clotting (TEG R, Fig 2A) and still yielded clots that were as strong (PEF A/high CaCl_2_) or stronger (PEF B/high CaCl_2_) than those produced by thrombin/high CaCl_2_ (Fig 2A). Thus, the strength of platelet gels formed with high CaCl_2_ and PEF A and B permits manipulation of the material and placement in position on a wound. Interestingly, TEG tracings of PRP treated with thrombin (but not PEF) showed evidence of thrombolysis leading to reduced clot strength which may make manipulation and application of the platelet gel to a wound more difficult. Clots failed to form when PRP supplemented with low CaCl_2_ (estimated 90 μM free Ca^2+^) was stimulated with PEF A or PEF B, making these conditions unsuitable for preparation of platelet gels for wound healing but allowing easy separation of released factors. However, as certain clinical procedures involve the injection of PRP [31–33], one could consider growth factor release with PEF via low CaCl_2_ with no clotting, and subsequent injection of the electrically stimulated PRP at the site of injury (*e*.*g*., elbow). Since thrombin activation results in growth factor release and clotting, some clinicians currently inject PRP that is not activated *ex vivo* [32,33] (because clotted PRP cannot be injected), with the hope that the collagen at the site of the injury will trigger PRP activation.

Prothrombin, the inactive precursor to thrombin, circulates in plasma at ~1.4 μM and is activated when Factor Xa cleaves the amino terminus, releasing the peptide fragment F1.2 [34]. Activation of platelets allows them to participate in conversion of prothrombin to thrombin by increasing surface exposure of negatively charged phospholipids, primarily phosphatidylserine, which allows binding of Xa and Ca^2+^, thereby greatly enhancing Xa’s prothrombinase activity.[35] Direct addition of thrombin to PRP results in approximately one-third as much F1.2, possibly as a result of thrombin combining with antithrombin which then inhibits Xa activity.[34] Previous investigations [36] suggest that addition of factor Xa to citrate anticoagulated plasma is much more efficient at generation of F1.2 than addition of thrombin. Therefore, the current results of higher levels of F1.2 in the presence of high CaCl_2_ alone compared to thrombin with low or high CaCl_2_ suggest that addition of CaCl_2_ without thrombin favors the generation of Xa, likely via the tissue factor pathway. PEF A and PEF B in the presence of high CaCl_2_ produce F1.2 levels higher than those produced by direct addition of bovine thrombin but lower than those produced by high CaCl_2_ alone, suggesting that Ca^2+^-mediated Xa production is less efficient in the presence of PEF A and PEF B but greater than that produced in the presence of low or high CaCl_2_ and thrombin.

### Differential release of platelet granules and growth factors

PEF A/low CaCl_2_ did not produce a clot, but it did result in a reduced number of residual alpha granules compared to saline treatment (Fig 7A, p = 0.044, unpaired t test) and a significant increase in the percentage of P-selectin-positive platelets (Fig 3A, Table 3), and significant release of PDI (Fig 3G, Table 3), PF4, PDGF, VEGF, and the highest level of released EGF (Fig 4, Table 4) of all conditions. In contrast, PEF B/low CaCl_2_ did not result a significant decrease in residual alpha granules (Fig 7A) or in a significant increase compared to vehicle in P-selectin, TLR9, PDI, or any of the growth factors (Tables 3 and 4). In PRP supplemented with high CaCl_2_, both PEF A and PEF B increased platelet surface exposure of the alpha granule marker P-selectin to a greater extent than high CaCl_2_ alone (Fig 3A and 3B) but to a lesser extent than thrombin/high CaCl_2_. The levels of PF4, PDGF, and VEGF, but not EGF, correlated with platelet alpha granule release as indicated by platelet surface P-selectin expression (MFI) and with T-granule release as indicated by platelet surface TLR9 [11] (Fig 5), while EGF levels, but not PF4, PDGF or VEGF, correlated with the level of PDI present in the supernatant after activation (Fig 5). Finally, supernatant concentrations of PF4, PDGF and VEGF, but not EGF, after activation were highly correlated with the residual number of alpha granules observed by TEM (Fig 7B–7D). Thus, the pattern of EGF release is distinct from that of PF4, PDGF and VEGF, suggesting that the distribution of EGF within platelets is also distinct from that of these other factors. Potential explanations for this novel correlation between released soluble PDI with soluble EGF include co-localization in selected platelet granules or localization in distinct granules whose release is triggered by similar stimuli. The enhanced release of EGF by PEF A is potentially useful in clinical situations, given that EGF is important for the epithelialization stage of wound healing [37,38].

Analysis of platelet morphology by TEM following PEF activation helped to address several important questions. First, the possibility that PEF causes the release of growth factors through mechanical fracturing of the platelets can be ruled out. Second, the observation that PEF A-activated platelets retain a large number of granules after PEF treatment, even in the presence of high CaCl_2_ provides support for the differences in growth factor levels observed by ELISA. Third, the distinct morphology of PEF A-activated platelets and distribution or remaining granules compared to thrombin-activated platelets suggests PEF initiates a process that is distinct from the thrombin activation pathway which has profound effects on the platelet cytoskeleton. Despite these differences, TEM shows that fibrinogen/fibrin fibers become closely associated with the PEF-activated platelets, suggesting a functional interaction with the adhesion receptor GPIIb-IIIa.

The pulse length for the PEF A condition used here (5 μsec monopolar pulse, ~4000 V/cm) is nearly 10 times longer than the conductive coupling pulses used in our previous studies.[12,13,15,16] PEF B (150 nsec bipolar pulse, ~4000 V/cm) is the same capacitive coupling pulse used in our recent work [15] (in [15] this was labeled as “Pulse A”). The longer pulses used here with PEF A, compared with previous studies could explain some of the findings. The impact of pulse duration on cells can generally be divided between submicrosecond pulses which generally have stronger effects on intracellular structures with less intense effects at the plasma membrane, while microsecond to millisecond pulses generally have stronger effects on the plasma membrane than on intracellular structures.[39–42] Thus, the large difference in EGF released with PEF A/low CaCl_2_ compared to practically no EGF release with PEF B/low CaCl_2_ may relate to effects of PEF A at the plasma membrane. Importantly, the pulse generator used for the present studies [16], in addition to reproducibly generating these pulses, also provides great flexibility to examine a wide variety of pulse durations, pulse shapes, and solution conductivities thereby permitting optimization of PRP activation for different applications.

## Conclusions

PEF conditions (*e*.*g*., pulse length, polarity) can be modulated to produce therapeutic platelet gels as strong or stronger those produced by thrombin, and this is tunable to produce growth factor profiles enhanced in specific factors important for different stages of wound healing. Differences in the levels of EGF in supernatants relative to other growth factors suggest EGF distribution within platelets is different from other growth factors. Moreover, the distinct morphology of PEF-treated platelets, and the pattern of growth factor release, suggests PEF initiates regulated release of platelet contents rather than non-specific mechanical fracturing.

## Supporting information

S1 Fig High resolution image corresponding to Fig 6A, left panel. See Fig 6 caption for details.
(JPG)

S2 Fig High resolution image corresponding to Fig 6A, right panel. See Fig 6 caption for details.
(JPG)

S3 Fig High resolution image corresponding to Fig 6B, left panel. See Fig 6 caption for details.
(JPG)

S4 Fig High resolution image corresponding to Fig 6B, right panel. See Fig 6 caption for details.
(JPG)

S5 Fig High resolution image corresponding to Fig 6C, left panel. See Fig 6 caption for details.
(JPG)

S6 Fig High resolution image corresponding to Fig 6C, right panel. See Fig 6 caption for details.
(JPG)

S7 Fig High resolution image corresponding to Fig 6D, left panel. See Fig 6 caption for details.
(JPG)

S8 Fig High resolution image corresponding to Fig 6D, right panel. See Fig 6 caption for details.
(JPG)
