# Supplementary figures and images for: Tunable activation of therapeutic platelet-rich plasma by pulse electric field: Differential effects on clot formation, growth factor release, and platelet morphology
Source: PLoS One. 2018 Sep 26;13(9):e0203557. doi: 10.1371/journal.pone.0203557 (PMC6157860; doi:10.1371/journal.pone.0203557)

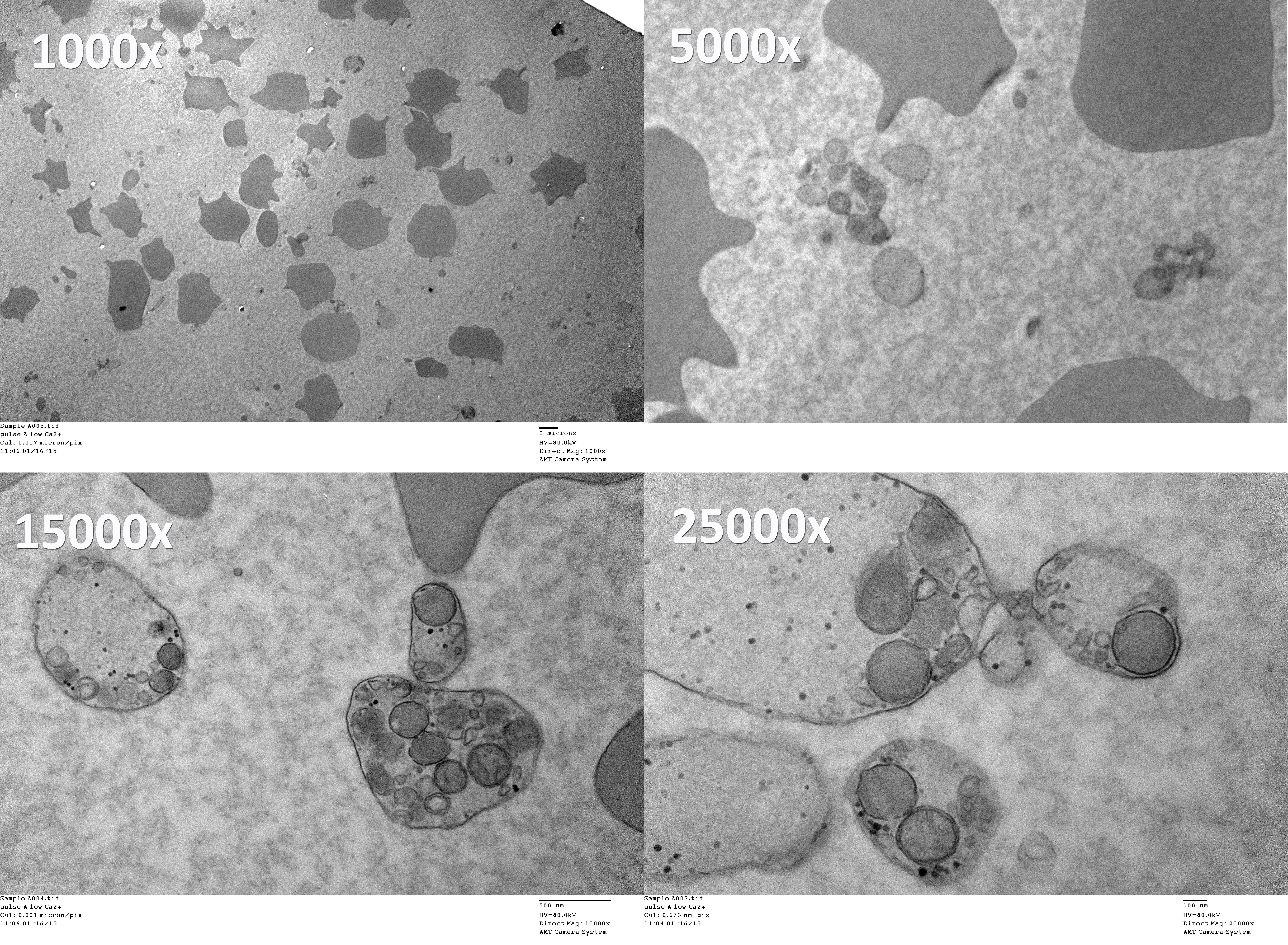

Supplement: S1 Fig — See Fig 6 caption for details. (JPG) [file pone.0203557.s001.jpg]

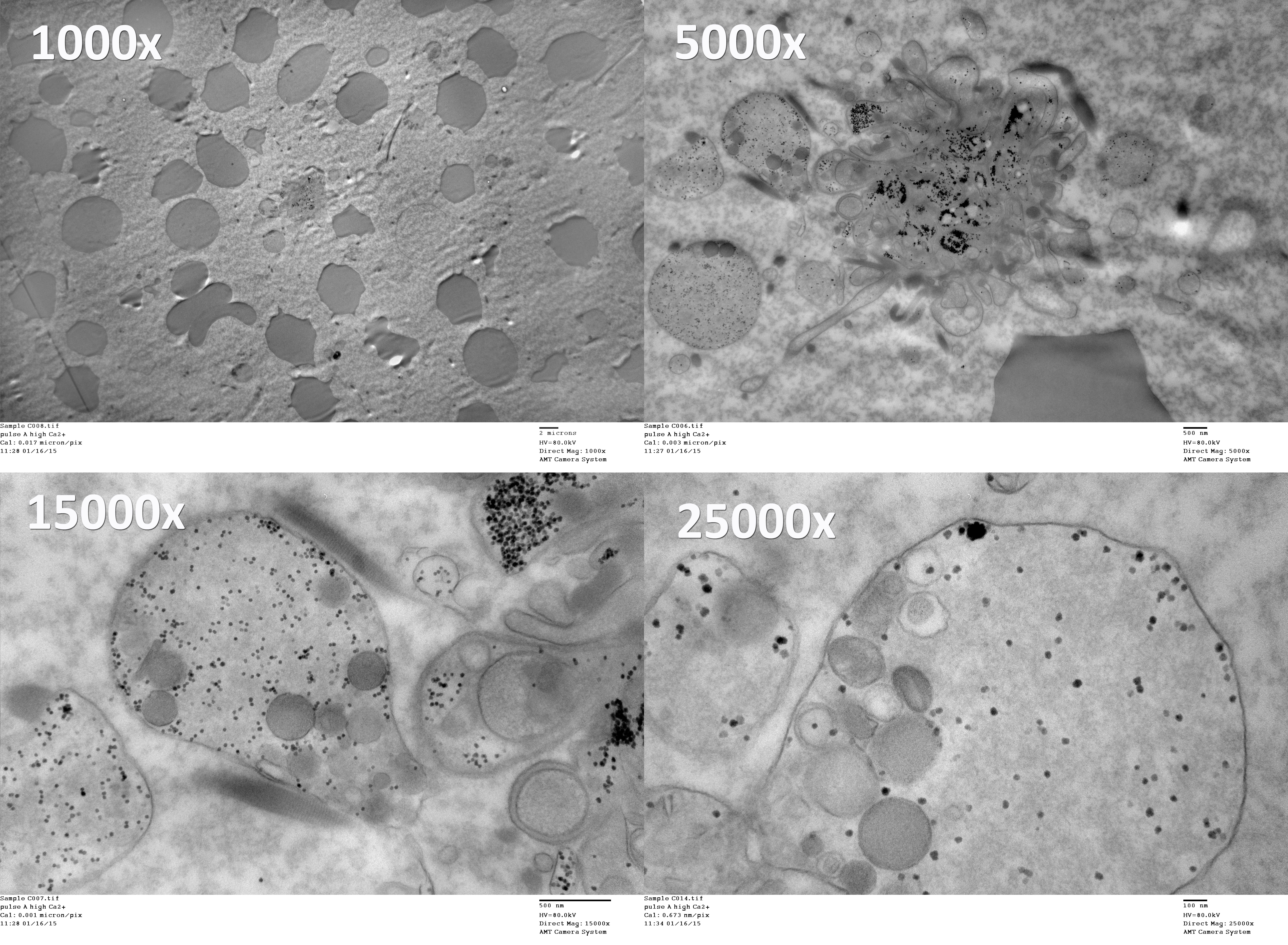

Supplement: S2 Fig — See Fig 6 caption for details. (JPG) [file pone.0203557.s002.jpg]

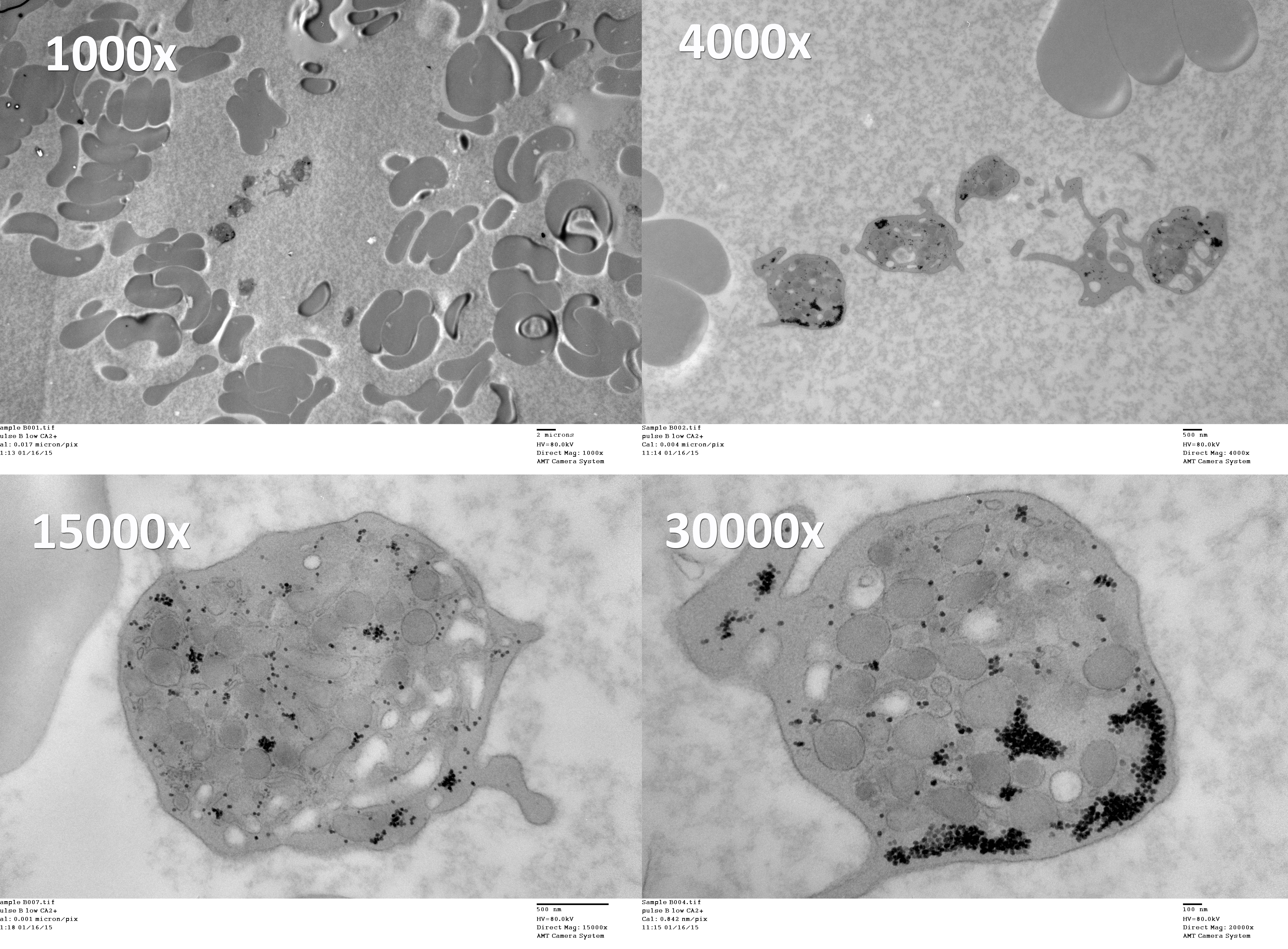

Supplement: S3 Fig — See Fig 6 caption for details. (JPG) [file pone.0203557.s003.jpg]

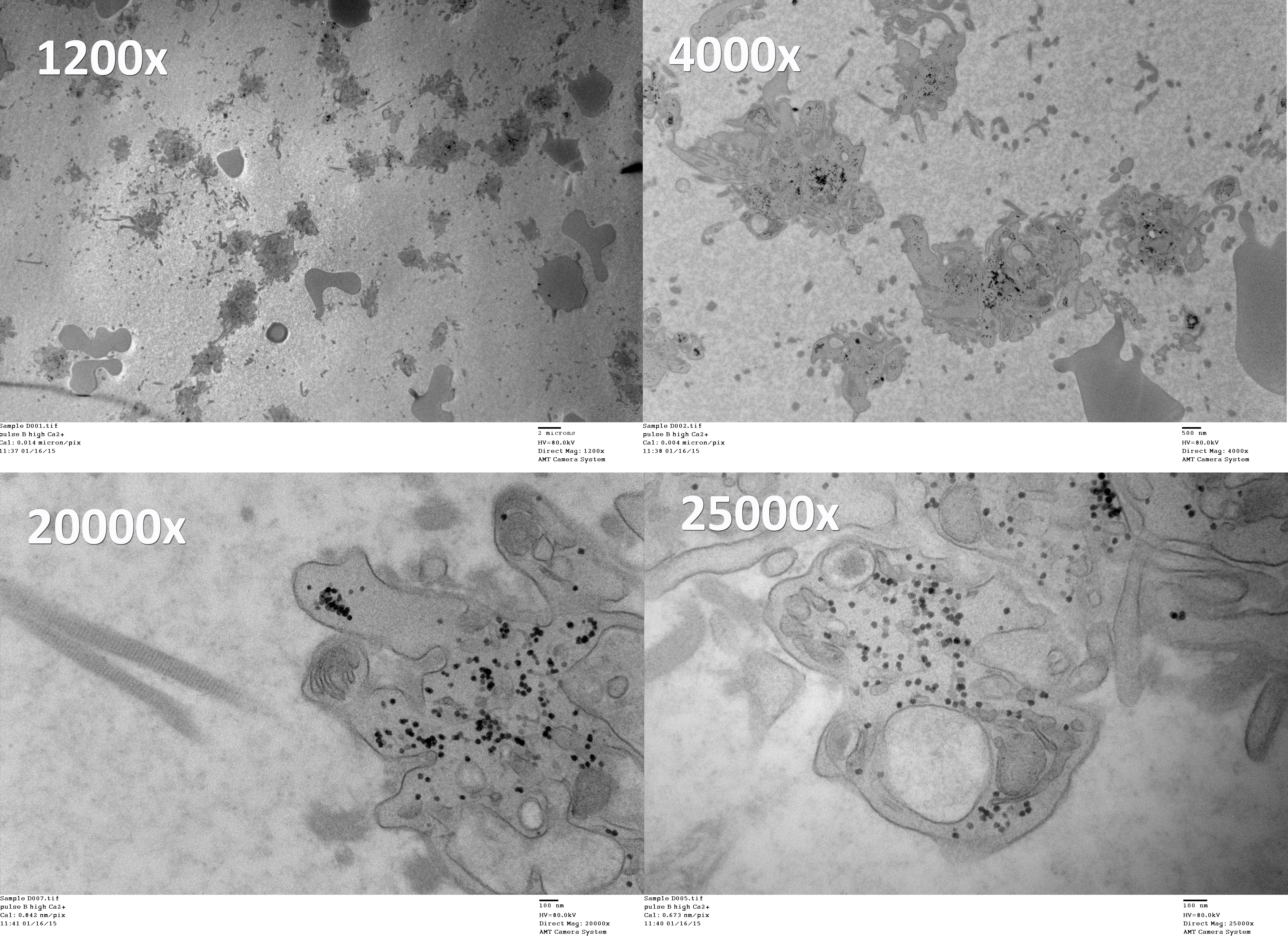

Supplement: S4 Fig — See Fig 6 caption for details. (JPG) [file pone.0203557.s004.jpg]

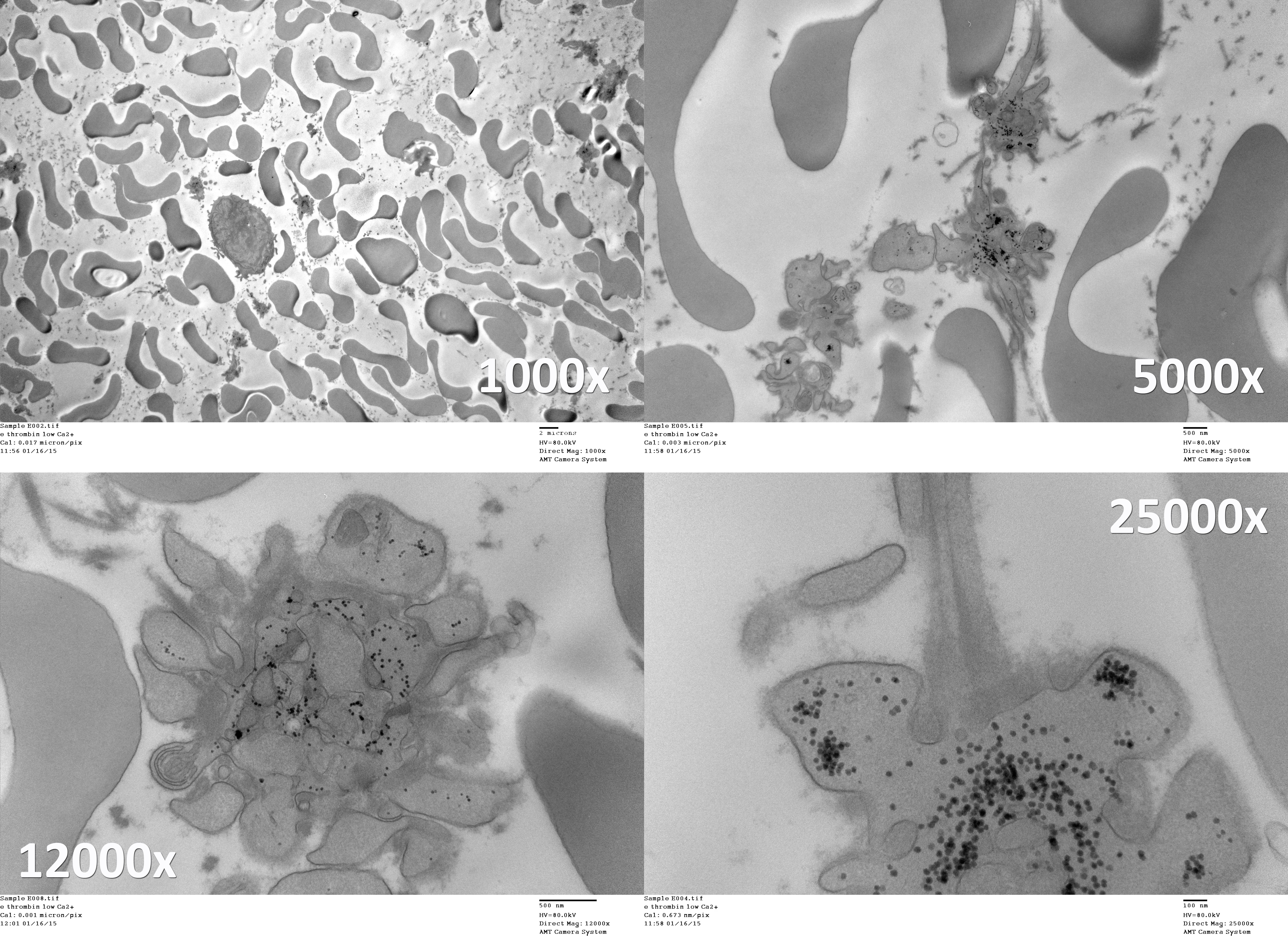

Supplement: S5 Fig — See Fig 6 caption for details. (JPG) [file pone.0203557.s005.jpg]

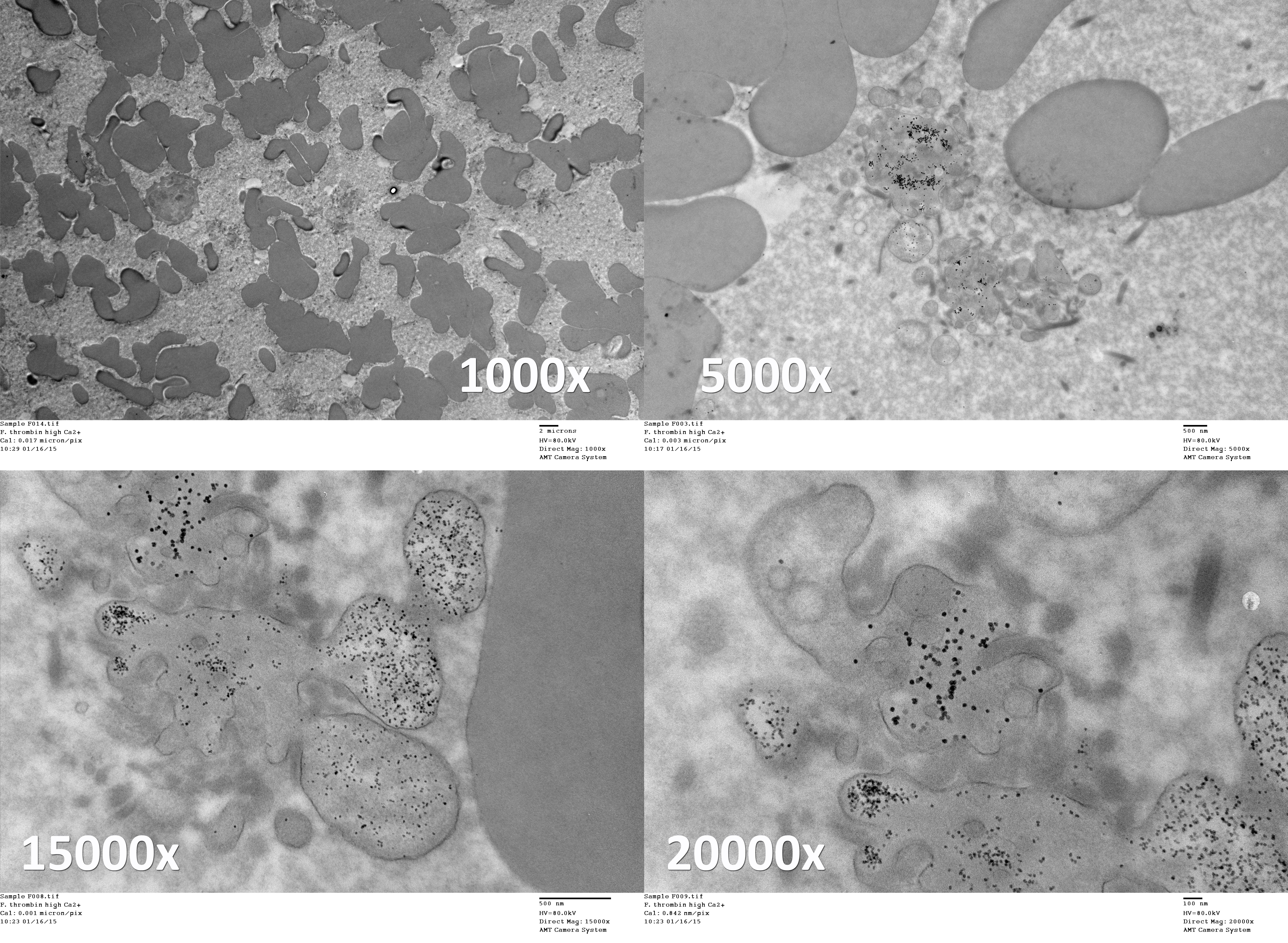

Supplement: S6 Fig — See Fig 6 caption for details. (JPG) [file pone.0203557.s006.jpg]

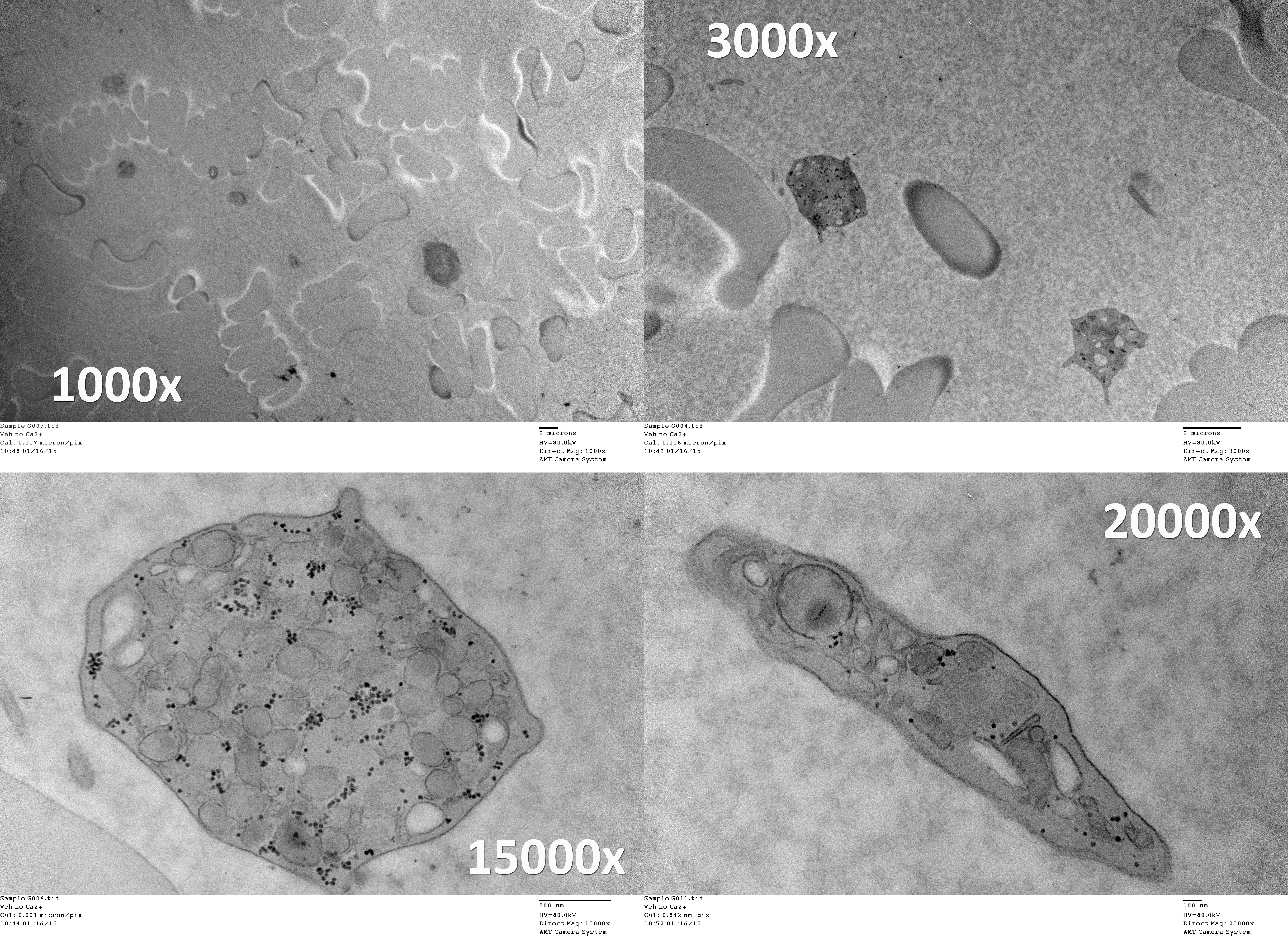

Supplement: S7 Fig — See Fig 6 caption for details. (JPG) [file pone.0203557.s007.jpg]

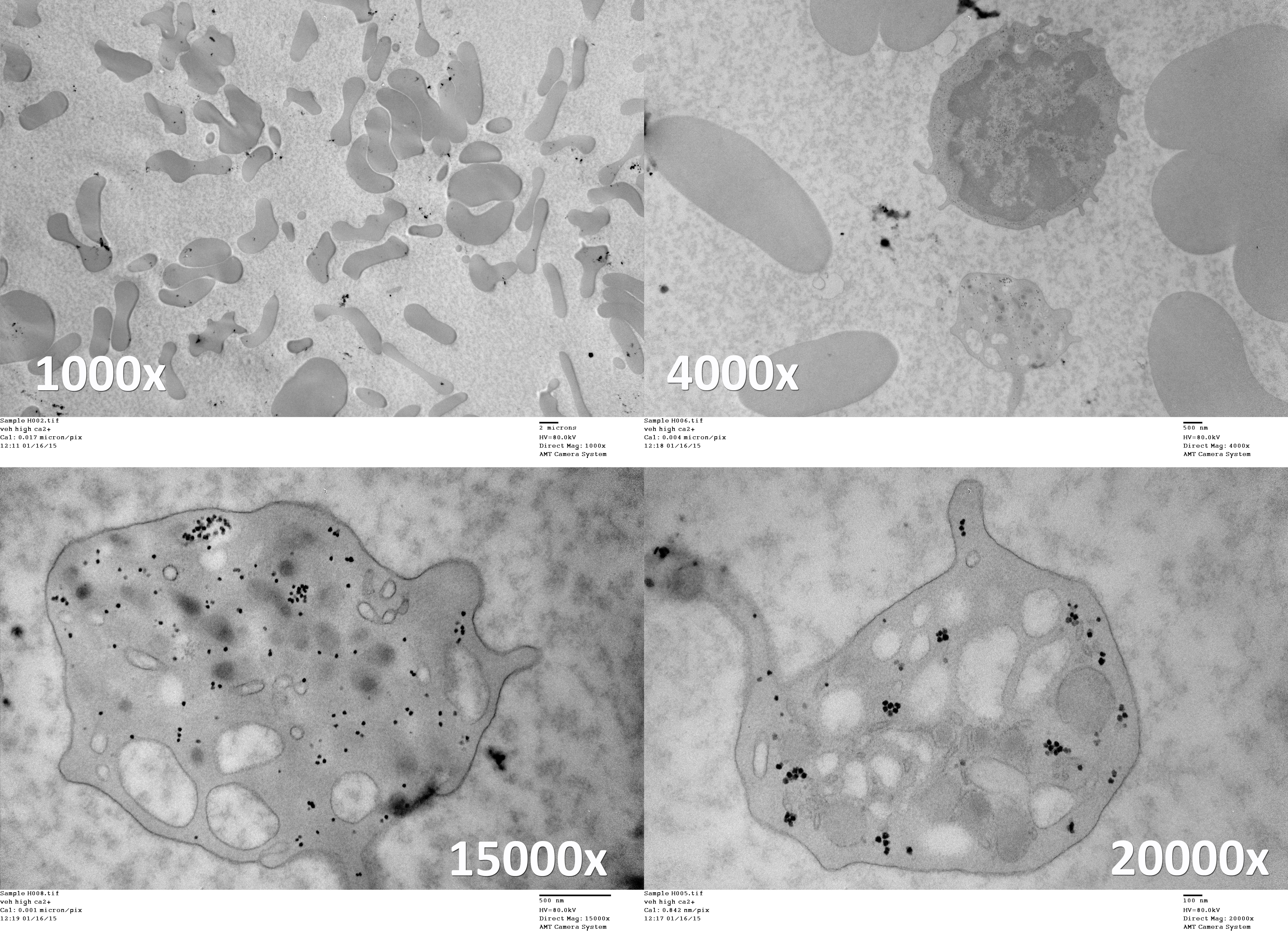

Supplement: S8 Fig — See Fig 6 caption for details. (JPG) [file pone.0203557.s008.jpg]
